# Supplementary material for: An in situ generated CAR-M with IFN-γ and negative dominance Sirpα isoform augments hepatocellular carcinoma immunotherapy
Source: J Nanobiotechnology. 2026 May 26;24:688. doi: 10.1186/s12951-026-04593-x (PMC13390385; doi:10.1186/s12951-026-04593-x)
Supplement: Supplementary file 1 — Supplementary material 1. [file 12951_2026_4593_MOESM1_ESM.docx]

**Supplementary figures and Figure legends**

**
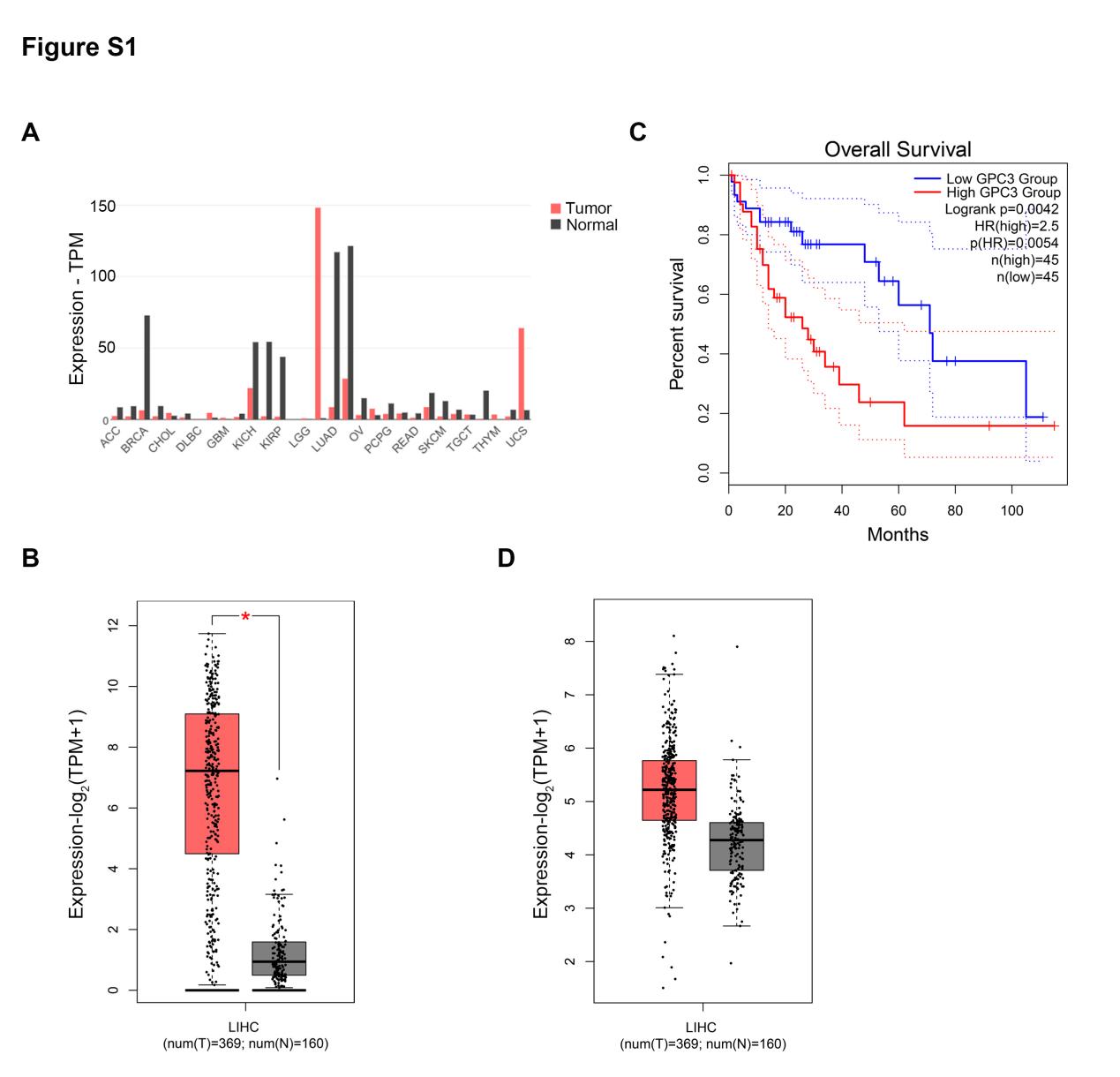
**

**Figure S1.** *GPC3* and *CD47* are highly expressed in liver hepatocellular carcinoma (LIHC). (A, B) *GPC3* is a relatively specific biomarker highly expressing in LIHC. Gene expression data were retrieved from the Gene Expression Profiling Interactive Analysis 2 (GEPIA2; http://gepia2.cancer-pku.cn/#index), an online platform based on The Cancer Genome Atlas (TCGA) and Genotype-Tissue Expression (GTEx) datasets. Bar heights indicate median expression levels in tumor tissues (T) or matched normal tissues (N); individual data points represent expression values from individual samples. All expression values were normalized using the log₂(TPM + 1) transformation, where TPM denotes Transcripts Per Million. (C) High *GPC3* expression correlates with reduced overall survival in LIHC patients. Survival analysis was conducted using the GEPIA2 platform with expression stratification at the 75th percentile (high vs. low) and results presented with 95% confidence intervals. (D) *CD47* is also highly expressed in LIHC patients. All data and images were obtained from the GEPIA online database. Gene expression data were retrieved from GEPIA2. Each data point corresponds to an individual sample.

**
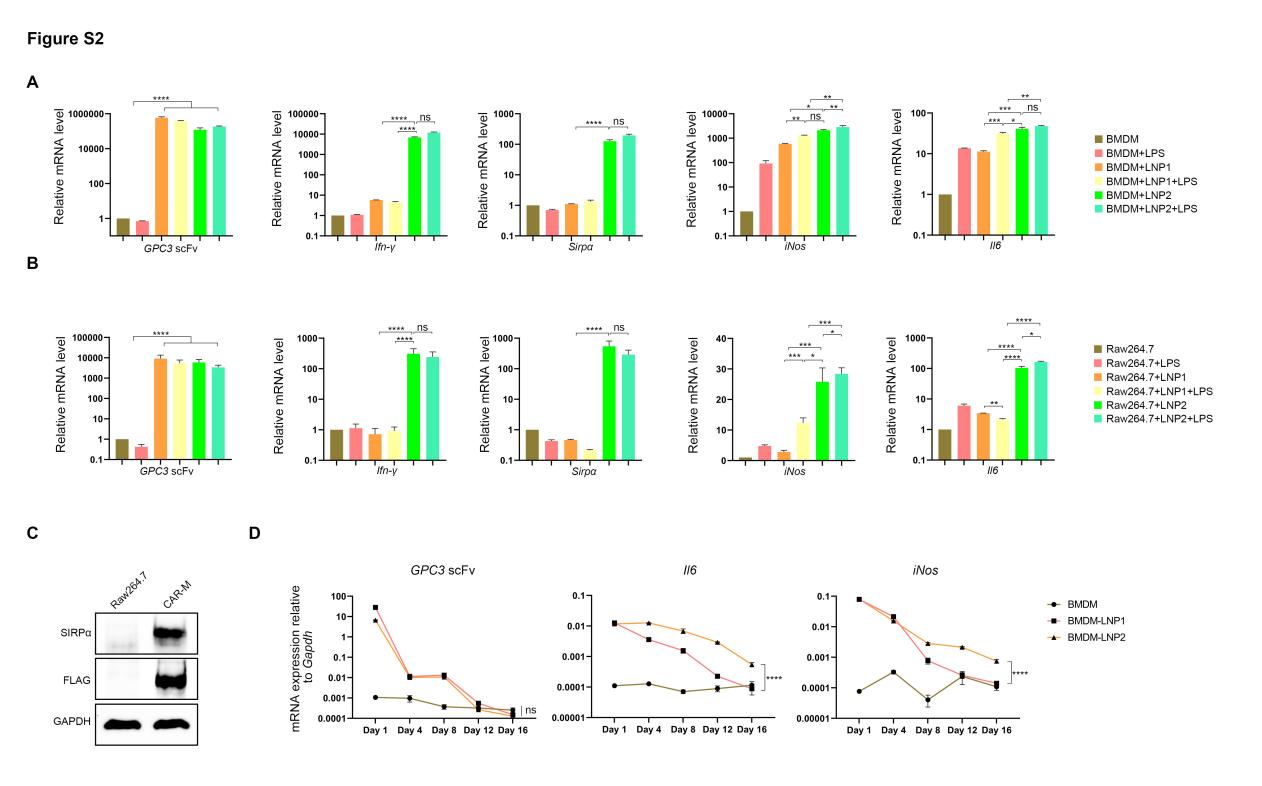
**

**Figure S2.** CAR-M cells armed with LNP1 or LNP2 exhibit an M1-polarized phenotype. (A, B) qRT-PCR analysis of CAR structural components and M1 macrophage marker gene expression in BMDM or Raw264.7 cells following treatment with LNP or LPS. Data are presented as mean ± s.e.m. from three independent experiments, and the significance was determined using two-tailed multiple t-tests analysis. **p* < 0.05; ***p* < 0.01; ****p* < 0.001; *****p* < 0.0001. ns, not significant. (C) Western blot detection of SIRPα protein and Flag tag in Raw264.7 and CAR-M cells. (D) Statistical analysis of qRT-PCR results over time. Day 0 was defined as the day of LNP1/2 transfection in BMDMs, with cells collected at regular intervals up to day 16. The experiment was repeated three times. The Y-axis represents mRNA expression levels normalized to GAPDH. Statistical significance was calculated using two-way ANOVA analysis. *****p* < 0.0001. ns, not significant.

**
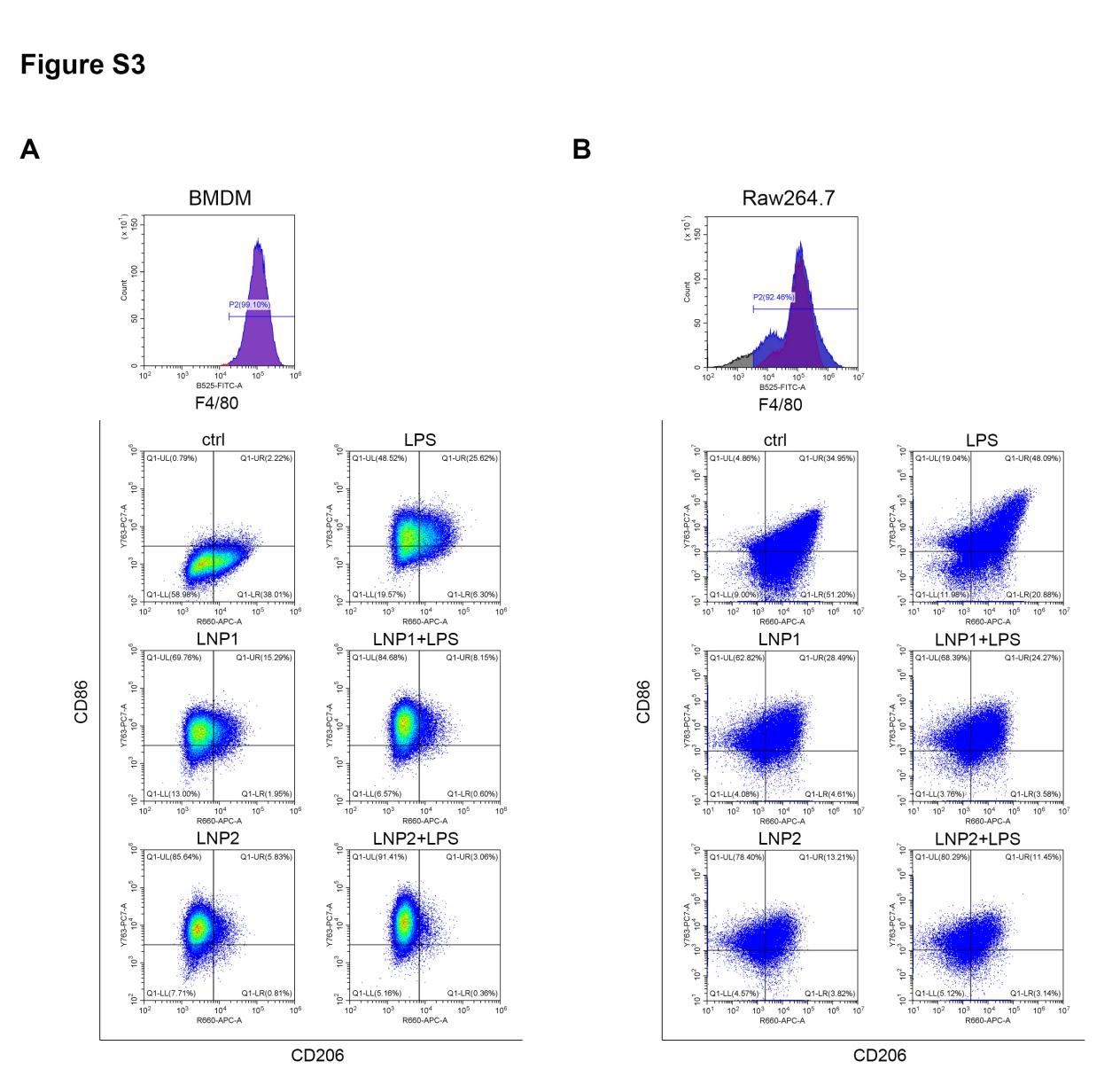
**

**Figure S3.** LNP1/2 transfection or LPS treatment promotes M1 polarization of macrophages. (A, B) Flow cytometric analysis of CD206 and CD86 expression in BMDM or Raw264.7 cells following treatment with LNP or LPS. CD86/CD206-positive cells were selected from the F4/80^+^ population.


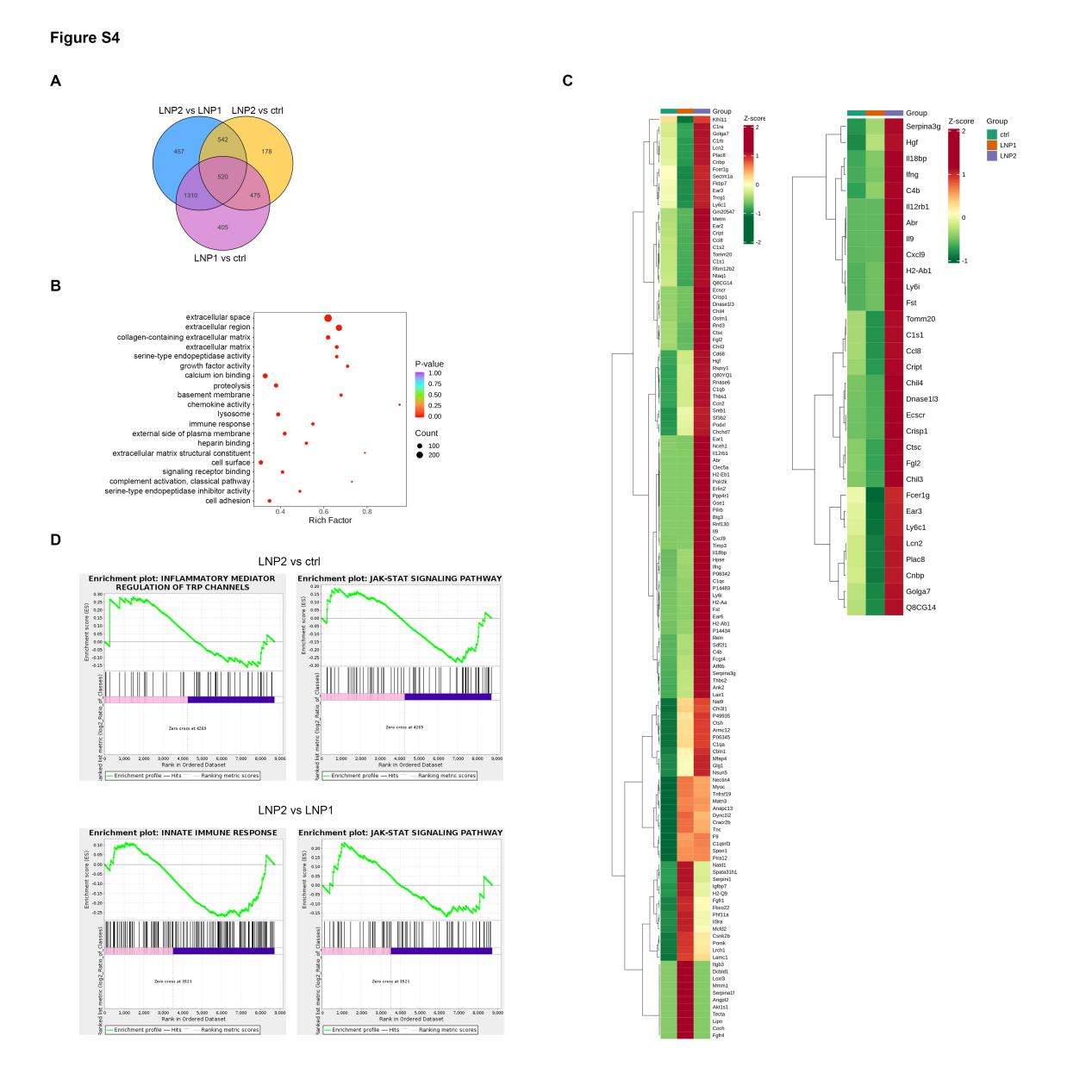


**Figure S4.** Mass spectrometry profiling of secretory proteins from BMDM cells armed with LNP1 or LNP2. (A) Venn diagram illustrating the overlap and differences in mass spectrometry results across groups. (B) Gene ontology (GO) analysis of DEPs among groups. (C) Heatmap of DEPs showing at least two-fold change between the LNP2 treatment group and other groups. The left panel highlights the top enriched secretory proteins in LNP2-armed CAR-M compared to the control group, while the right panel shows those highly enriched in LNP2-armed CAR-M relative to LNP1-armed CAR-M. (D) GSEA analysis of DEPs comparing LNP2 with control or LNP2 with LNP1 treatment groups.

**
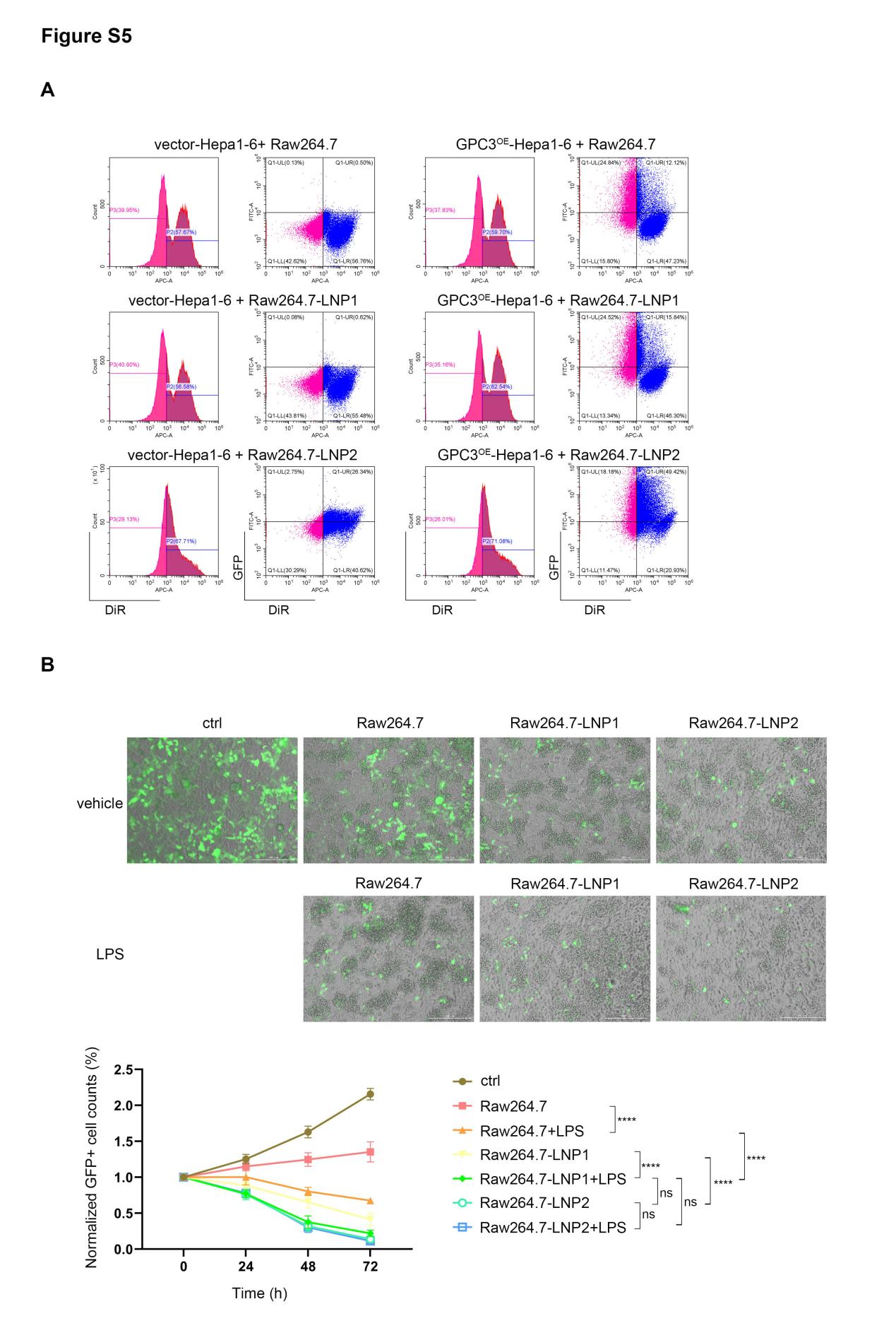
**

**Figure S5.** CAR-M cells demonstrate potent anti-tumor activity in vitro. (A) Flow cytometric results showing DiR^+^ or GFP^+^ cells after 6 hours of co-culture between Hepa1-6 and Raw264.7 cells. The left panel displays results from control Hepa1-6 cells transfected with a GFP vector, while the right panel shows results from GPC3-2A-GFP-overexpressing Hepa1-6 cells. (B) Live-cell imaging of GPC3^OE^-Hepa1-6 cells after 72 hours of co-culture with CAR-M cells at a 1:1 E:T ratio, along with time-course quantification of GFP^+^ cells across treatment groups. The statistical data were presented as mean ± s.e.m., and two-way ANOVA analysis was used to calculate the significance. *****p* < 0.0001. ns, not significant.

**
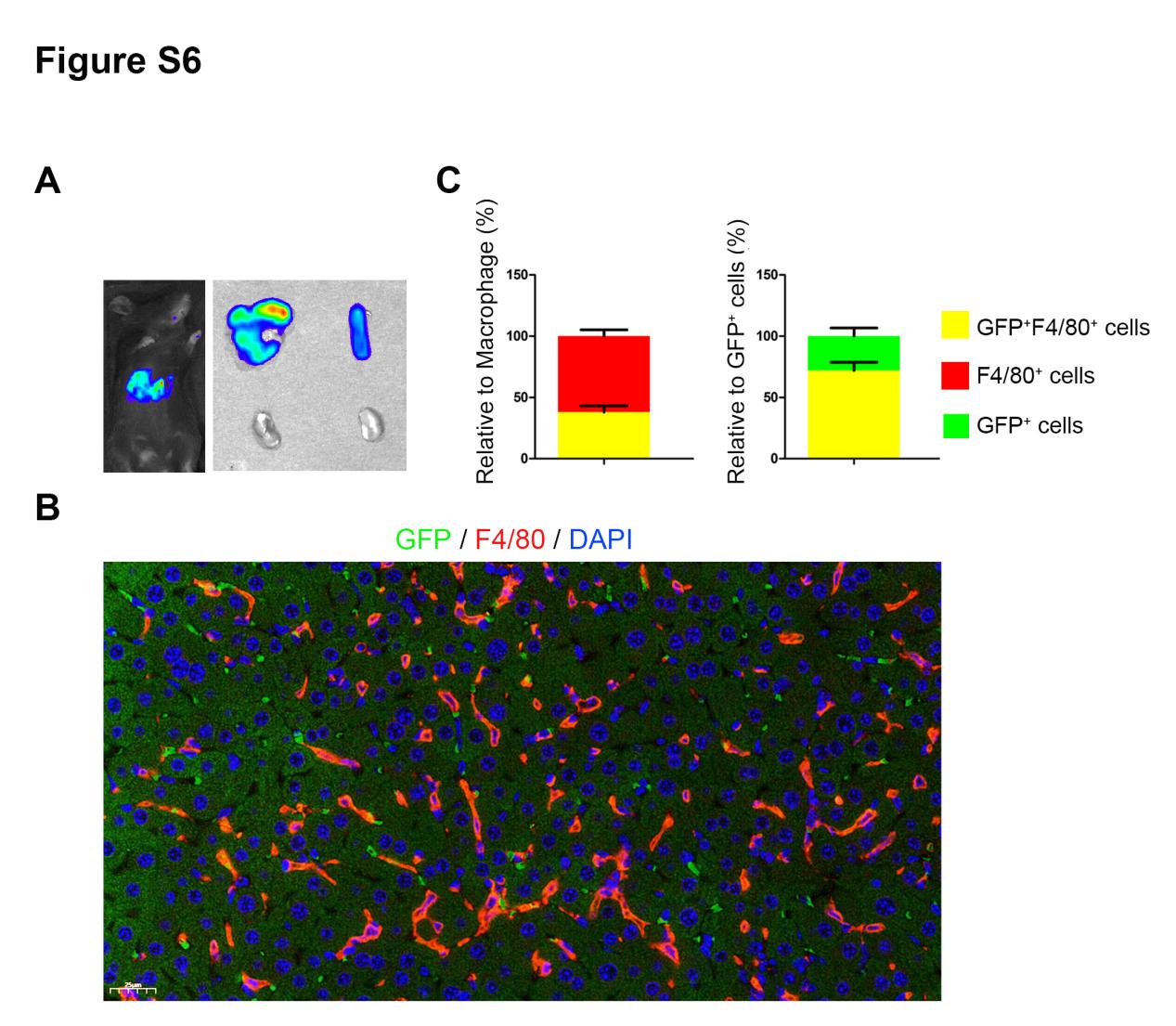
**

**Figure S6.** Intravenous injection of LNP targets the liver in mice in vivo. (A) Bioluminescence signal of mice 4 hours after intravenous injection of LNP-luciferase. The dosage of LNP-mRNA was approximately 5 μg diluted in 200 μL of PBS solution. The right panel shows the distribution of the signal across different organs, including the liver, spleen, and kidney. (B) Immunofluorescence image of GFP signals in mice liver tissues after intravenous injection of LNP-eGFP. The liver tissues were dissected, fixed, paraffin-embedded, sectioned, and then subjected to immunofluorescence labeling of F4/80 cells. The spatial relationship between GFP-expressing cells and Kupffer cells/macrophages was subsequently analyzed. (C) Stacked bar chart showing the proportion of GFP^+^ F4/80^+^ cells among total GFP^+^ cells or total F4/80^+^ cells. Fluorescence signals were quantified using ImageJ software.


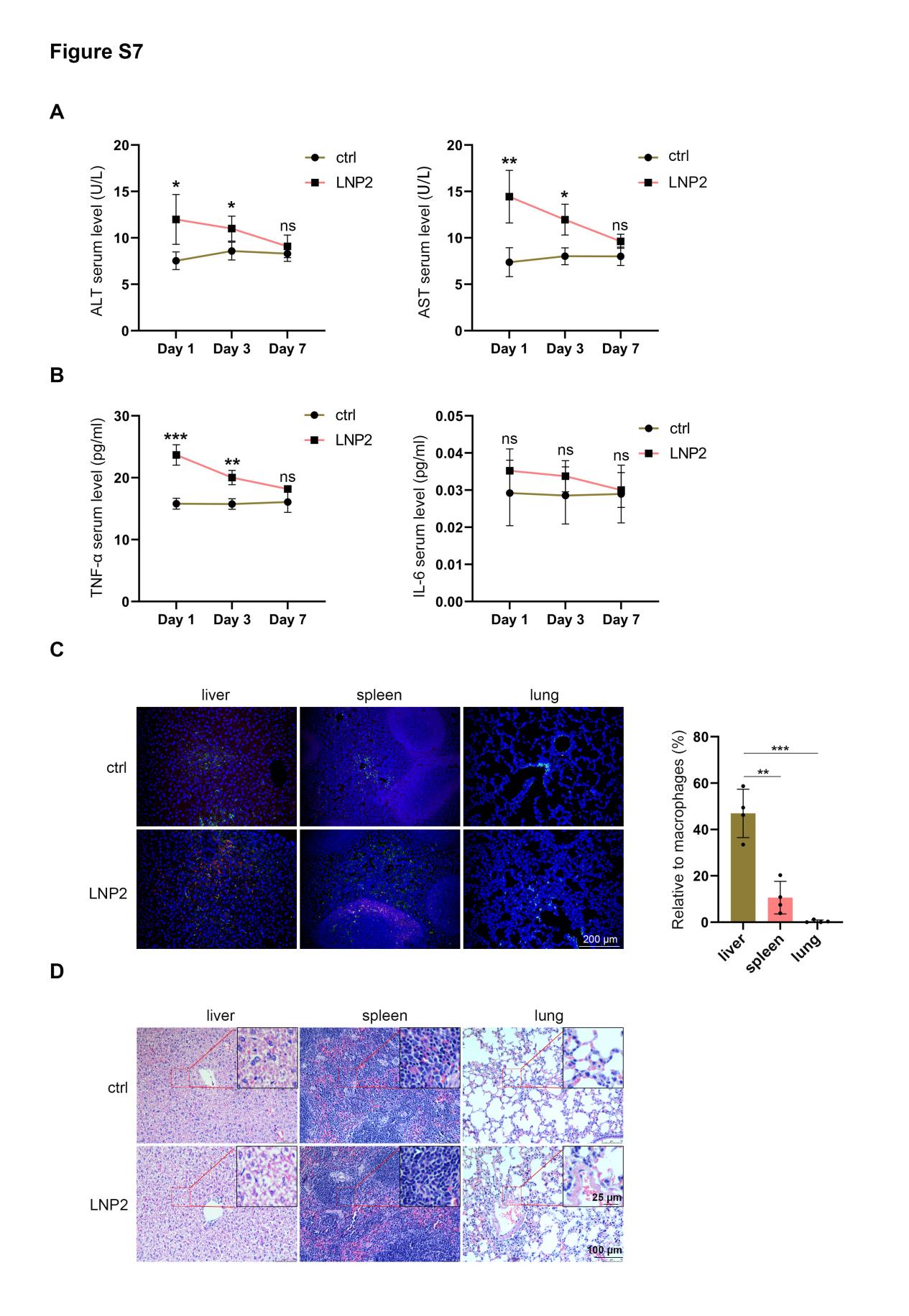


**Figure S7.** In vivo safety analysis of LNP2 in mice. (A, B) Serum concentrations of hepatocellular enzymes and pro-inflammatory cytokines in C57BL/6 mice following LNP2 administration. Blood was collected via tail vein puncture at designated time points, and analytes were quantified using commercially available enzymatic assay kits (ALT/AST) or ELISA kits (cytokines), respectively. (C) Immunofluorescence examination of Flag-tagged CAR (red) and F4/80 (green) in liver, spleen, and lung organ in mice 24 hour after LNP2 injection. (D) H&E staining of matched tissue sections, showing preserved microarchitecture across all three organs. A high magnification inset has been incorporated into the upper right corner of every panel to facilitate morphological assessment. Data were presented as mean±sem., and significance was evaluated by t-test analysis. **p* < 0.05; ***p* < 0.01; ****p* < 0.001; ns, not significant.


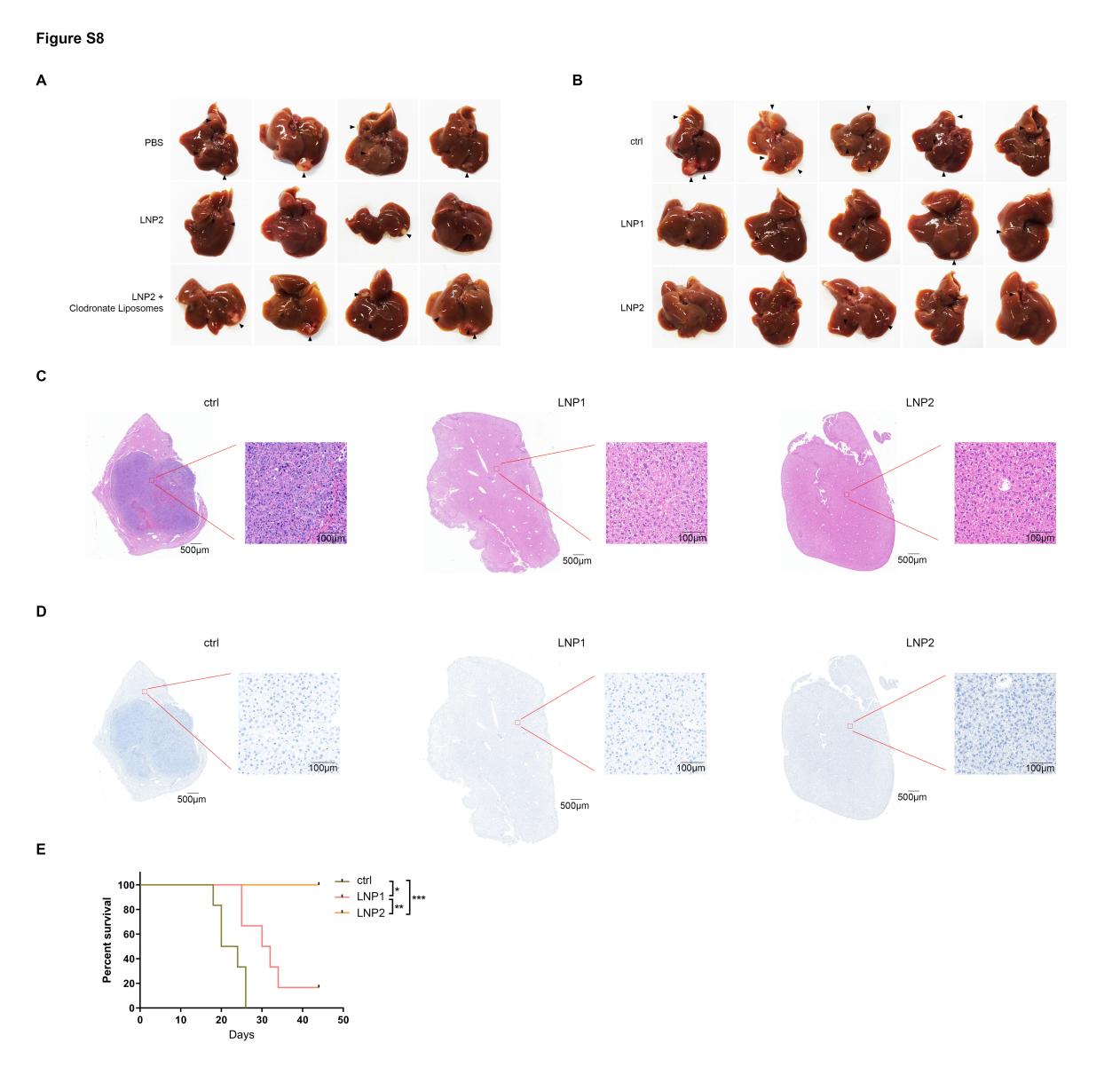


**Figure S8.** CAR-Ms generated by in vivo LNP delivery suppress tumor growth in the mouse liver. (A, B) Dissected liver organs from orthotopic xenograft tumor-bearing mice after administration of LNP1/2 or clodronate liposomes. Black arrows indicate tumor lesions in the liver. (C, D) Representative images of H&E and p-STAT1 immunohistochemical staining from liver tissues of the aforementioned mouse model are shown. The rectangle indicates a locally magnified area. In the control group, both the tumor region and its surrounding microenvironment were visible within the same field of view. p-STAT1 expression was specifically detected in the tumor microenvironment. (E) Kaplan-Meier survival analysis of liver orthotopic tumor model mice with or without LNP1/2 administration. Day 0 corresponds 10 days after tumor implantation.


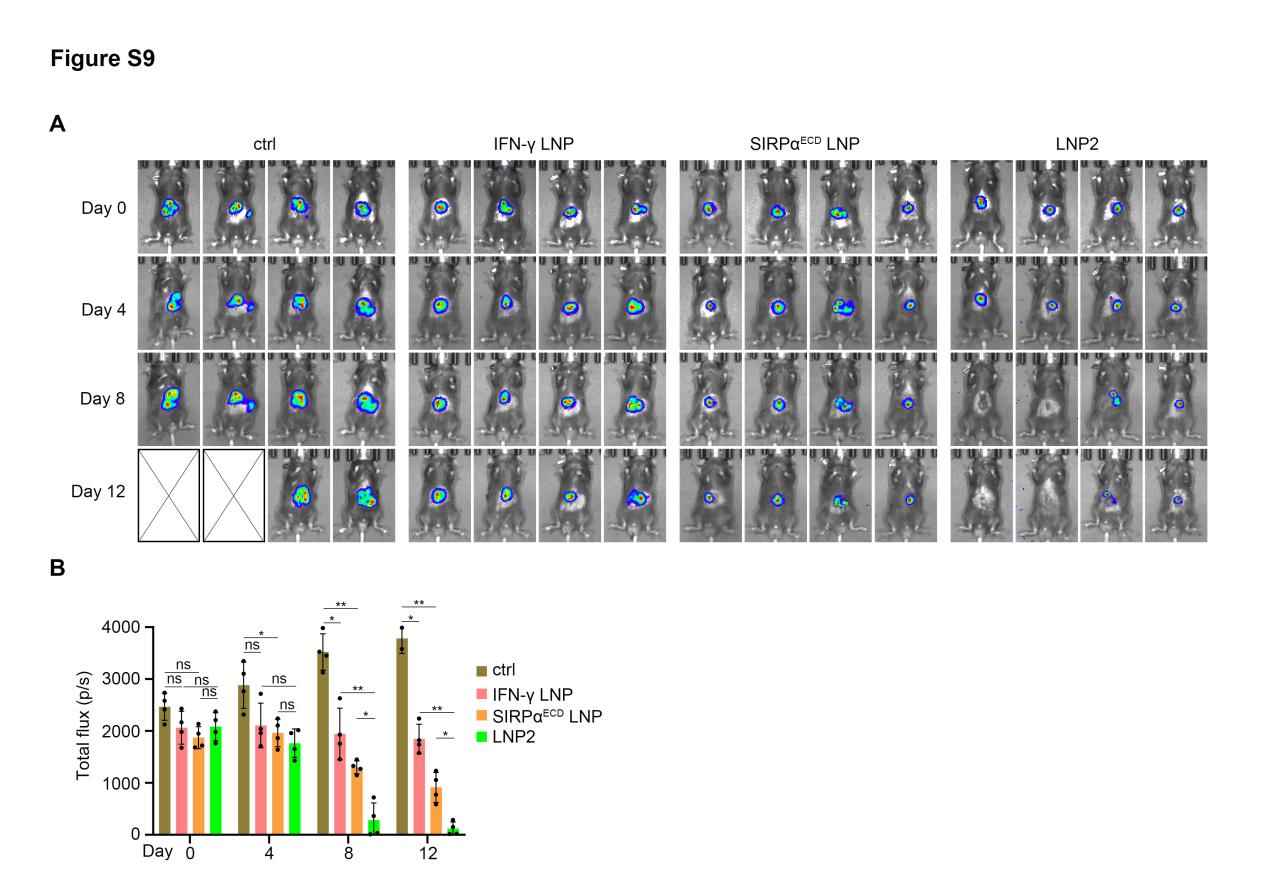


**Figure S9.** Therapeutic evaluation of LNP-mRNA drugs in murine orthotopic HCC model. (A) In vivo bioluminescence imaging showing time-dependent tumor growth in mice following treatment with LNPs. (B) The quantification of bioluminescence signal across different treatment groups over time. Data were presented as mean±sem., and significance was evaluated by t-test analysis. **p* < 0.05; ***p* < 0.01. ns, not significant.


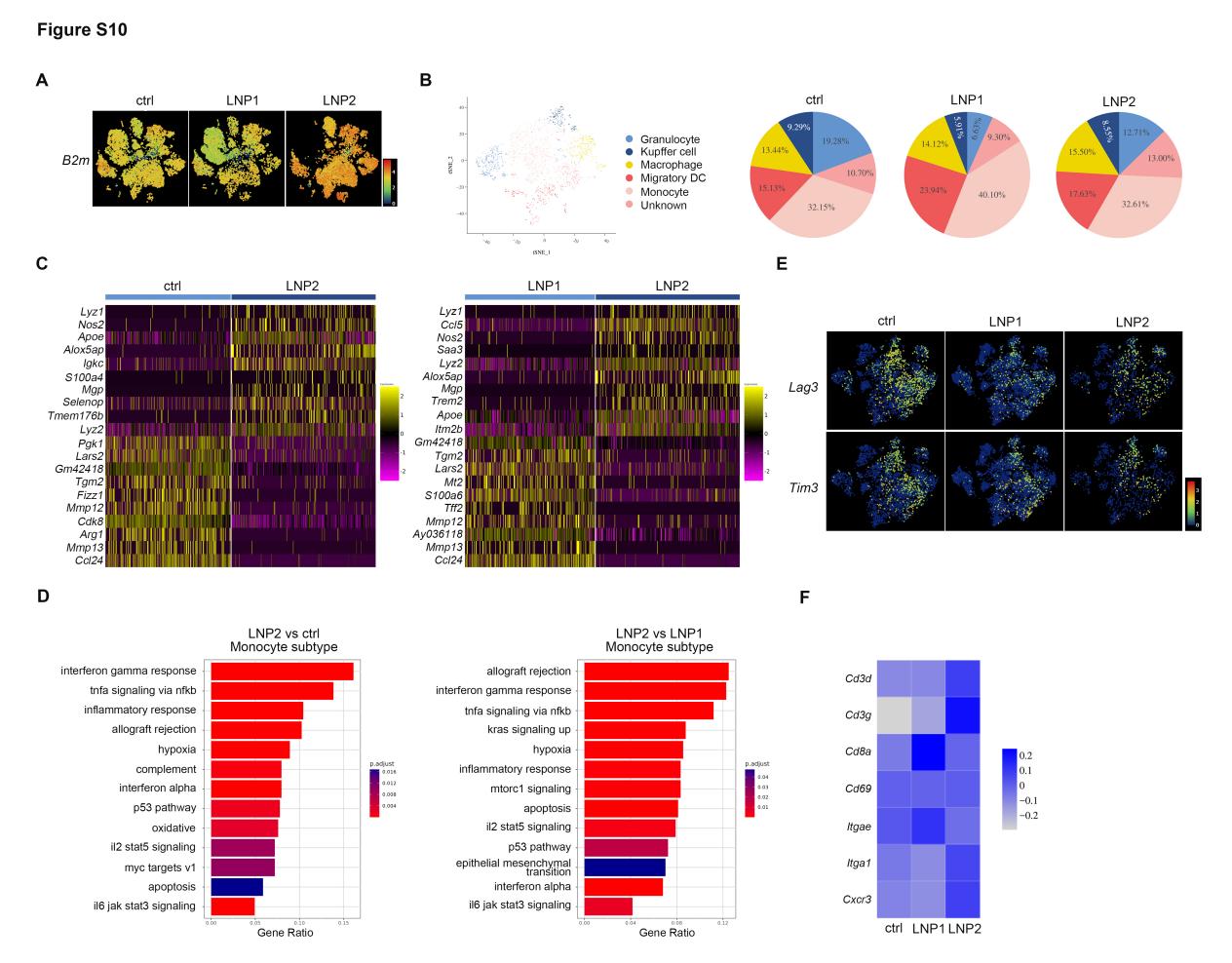


**Figure S10.** LNP2-armed CAR-Ms remodel the immune microenvironment into a pro-inflammatory and anti-tumor state. (A) mRNA expression levels of *B2m* in the TME across different samples, with color intensity representing gene expression levels. (B) t-SNE plot and pie chart depicting the distribution of cell subsets within the monocyte group across different samples. (C) Heatmap analysis showing the most enriched genes in the monocyte group from LNP2-treated mice compared to control or LNP1-treated mice. (D) Bar plot displaying the most significantly enriched Hallmark gene sets differentially expressed in the monocyte group between LNP2 and control or LNP2 and LNP1-treated mice. Hallmark gene sets were obtained from the MSigDB database. (E) mRNA expression levels of *Lag3* and *Tim3* in the TME across different samples, with color intensity reflecting gene expression levels. (F) Heatmap analysis of the expression of effector memory T cell-related genes across different samples.


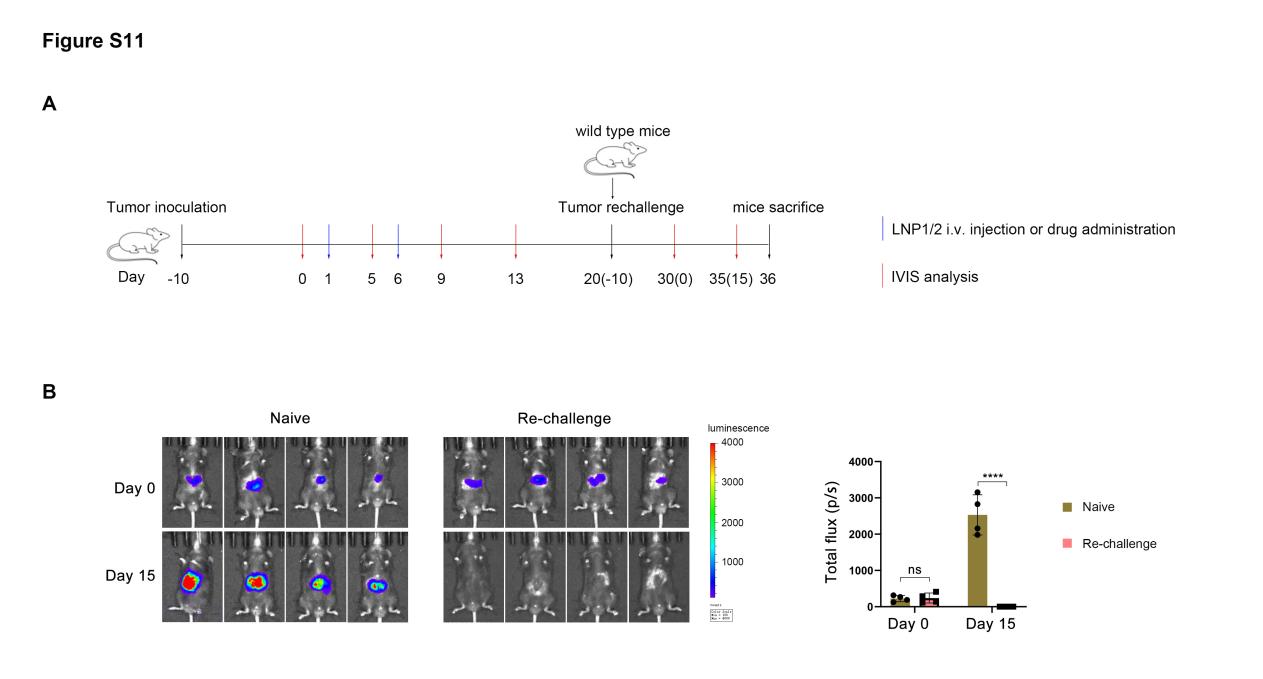


**Figure S11.** LNP2 pre-treatment enables mice to establish immune memory against GPC3-overexpressing tumor cells. (A) Schematic illustration of the experimental design for the tumor re-challenge assay in a liver orthotopic xenograft tumor model. (B) In vivo bioluminescence imaging showing time-dependent tumor growth in mice across different treatment groups. The right panel presents the quantification of bioluminescence signals across treatment groups.

**
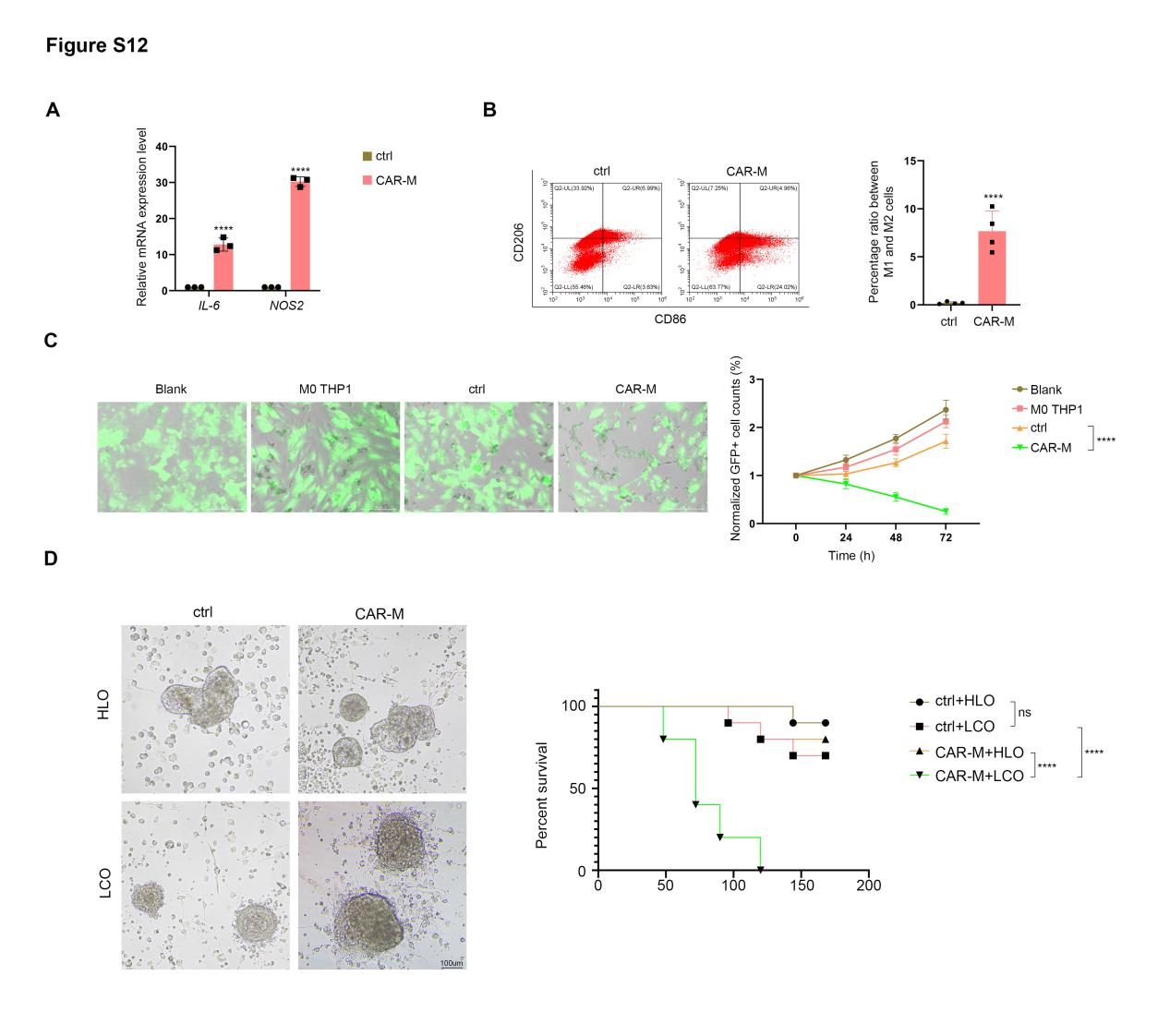
**

**Figure S12**. CAR-Ms produced by LNP transfection efficiently eliminate human liver cancer cells in vitro. (A) qRT-PCR detection of M1 marker expression in PBMC-derived macrophages (ctrl) or CAR-Ms. (B) Flow cytometric evaluation of CD86 and CD206 expression in control macrophages or CAR-Ms, along with the corresponding quantification of M1/M2 ratios. (C) Live-cell imaging of GFP-overexpressing Huh7 cells after 72 hours of co-culture with THP1 or CAR-M cells. No THP1 cells were added in the blank group. M0 THP1 cells refer to THP1 cells without activation factor treatment, whereas the ctrl group refers to THP1 cells pre-treated with LPS, IFN-γ and empty LNP. The right panel shows the time-course quantification of GFP^+^ cells across treatment groups. (D) Morphological dynamics of HLO or LCO co-cultured with control or THP1-derived CAR-M cells over time. The organoids shown are representative of at least 30 organoids per group. The right panel presents Kaplan-Meier survival analysis of HLO or LCO after long-term co-culture with THP1 control or CAR-M cells. The co-culture system was observed and recorded every 24 hours. Organoids exhibiting apparent disaggregation or disruption were considered as death events. Statistical data were presented as mean ± s.e.m., and t-tests analysis or two-way ANOVA analysis was used to calculate the significance. *****p* < 0.0001. ns, not significant.
